# Serum LDH: a potential surrogate to chest radiograph in pediatric Covid-19 patients to reduce radiation exposure

**DOI:** 10.1186/s43055-022-00805-0

**Published:** 2022-06-07

**Authors:** Karuna M. Das, Jamal Aldeen Alkoteesh, Usama M. AlBastaki, Rajvir Singh, Abbey J. Winant, Anisha P, Amrita Das, Klaus Van Gorkom, Edward Y. Lee

**Affiliations:** 1 Department of Radiology, CMHS, UAEU, Al Ain, United Arab Emirates; 2 Tawam Hospital, Al Ain, United Arab Emirates; 3 Department of Radiology, Rashid Hospital, Dubai, United Arab Emirates; 4 Department of Biostatistics, AIIMS, New Delhi, India; 5 Department of Radiology, Boston Children’s Hospital, Harvard Medical School, Boston, USA; 6 Department of Biostatistics, Manipal Medical College, Manipal, India; 7 Brighton College, Al Ain, United Arab Emirates

**Keywords:** COVID-19, Children, LDH, Chest radiograph, Radiation dose

## Abstract

**Background:**

Chest radiographs are frequently used to evaluate pediatric patients with COVID-19 infection during the current pandemic. Despite the minimal radiation dose associated with chest radiography, children are far more sensitive to ionizing radiation's carcinogenic effects than adults. This study aimed to examine whether serum biochemical markers could be potentially used as a surrogate for imaging findings to reduce radiation exposure.

**Methods:**

The retrospective posthoc analysis of 187 pediatric patients who underwent initial chest radiographs and serum biochemical parameters on the first day of emergency department admission. The cohort was separated into two groups according to whether or not the initial chest radiograph revealed evidence of pneumonia. Spearman's rank correlation was used to connect serum biochemical markers with observations on chest radiographs. The Student's t-test was employed for normally distributed data, and for non-normally distributed data, the Mann–Whitney U test was used. A simple binary logistic regression was used to determine the importance of LDH in predicting chest radiographs. The discriminating ability of LDH in predicting chest radiographs was determined using receiver operating characteristics (ROC) analysis. The cut-off value was determined using Youden's test. Interobserver agreement was quantified using the Cohen k coefficient.

**Results:**

187 chest radiographs from 187 individual pediatric patients (95 boys and 92 girls; mean age ± SD, 10.1 ± 6.0 years; range, nine months–18 years) were evaluated. The first group has 103 patients who did not have pneumonia on chest radiographs, while the second group contains 84 patients who had evidence of pneumonia on chest radiographs. GGO, GGO with consolidation, consolidation, and peri-bronchial thickening were deemed radiographic evidence of pneumonia in group 2 patients. Individuals in group 2 with radiological indications of pneumonia had significantly higher LDH levels (*p* = 0.001) than patients in group 1. The Spearman's rank correlation coefficient between LDH and chest radiography score is 0.425, showing a significant link. With a *p*-value of < 0.001, the simple binary logistic regression analysis result validated the relevance of LDH in predicting chest radiography. An abnormal chest radiograph was related to LDH > 200.50 U/L (AUC = 0.75), according to the ROC method. Interobserver agreement between the two reviewers was almost perfect for chest radiography results in both groups (*k* = 0.96, *p* = 0.001).

**Conclusion:**

This study results show that, compared to other biochemical indicators, LDH has an 80.6% sensitivity and a 62% specificity for predicting abnormal chest radiographs in a pediatric patient with confirmed COVID-19 infection. It also emphasizes that biochemical measures, rather than chest radiological imaging, can detect the pathogenic response to COVID-19 infection in the chest earlier. As a result, we hypothesized LDH levels might be potentially used instead of chest radiography in children with COVID-19, reducing radiation exposure.

## Background

Children have been substantially affected by the coronavirus disease (COVID-19), with about 4.3 million tests positive for the virus since the pandemic, accounting for 1.5–3.5% of their hospitalizations and 0.00–0.03% of fatalities [1, 2]. Imaging studies, such as chest radiography and computed tomography (CT), are frequently used with quantitative reverse transcriptase-polymerase chain reaction (RT-PCR) tests for the initial diagnosis and evaluation of disease progression in pediatric COVID-19 pneumonia [3–6]. Unfortunately, these imaging studies are associated with ionizing radiation exposure, which may be harmful to the vulnerable pediatric population [6–9]. The average effective dose for chest radiographs taken in AP, PA, and lateral projection is 0.14, 0.07, and 0.22 mSv, respectively [10]. While a single chest radiograph may have minimal radiation, pediatric patients require over one radiograph to monitor their health, resulting in reasonably large cumulative doses [11–13]. Without a threshold, cancer risk increases linearly with decreasing doses, and even the smallest dose can cause a moderate increase in risk in humans [12, 13]. Therefore, there is an urgent need for a potential surrogate test that can be used instead of chest radiographs to diagnose and evaluate COVID-19 infection in children.

COVID-19 is a systemic infection that, although incompletely understood, has a substantial pathologic impact on multiple organ systems throughout the body, leading to abnormalities in serum biochemical parameters. Several recent publications have reported altered serum biochemical parameters in pediatric and adult populations [14–16]. Lactate dehydrogenase (LDH) was recently identified as one of the most common biochemical parameters in COVID-19-infected children. It was deemed a prospective predictor of serum biochemical changes in the bodies of COVID-19-infected children [17]. LDH was found to be significantly high in two recent studies, where they compared the biochemical parameter with that of CT and chest radiographs in COVID-19 in children [18–20].

None of these studies directly correlated LDH with that chest radiography findings to the best of our knowledge. Therefore, this study examined the relationship between chest radiographic findings and serum biochemical parameters in pediatric patients with COVID-19 by directly comparing groups with and without chest radiographic findings and determining whether serum biochemical parameters can be potentially surrogates for chest radiography.

## Methods

### Institutional review board approval

Our institutional review board approved this retrospective study to review chest radiographs, serum biochemical parameters, and patient outcomes. A waiver for informed consent was obtained. Patient confidentiality was protected by closely following the Health Insurance Portability and Accountability Act guidelines.

The radiology department information system identified all consecutive pediatric patients (less than or equal to18 years old) diagnosed with COVID-19 from March 2020 to December 2020. The patient inclusion criteria were as follows: (a) COVID-19 confirmation by RT-PCR test from respiratory secretions using a nasopharyngeal or oropharyngeal swab based on the criteria set by the Centers for Disease Control [21] and (b) availability of both chest radiographs and serum biochemical parameters on the first day of arrival at the hospital. The final study group included 187 children who had chest radiographs and serum biochemical measurements taken on the first day of their emergency department stay. According to the department of health policy, all pediatric patients who tested positive for COVID-19 (RT-PCR) were isolated and quarantined for 15 days in adjacent hotel premises, including days spent in the emergency room.

Of note, 56 (29.9%) of the 187 chest radiographs included in our study were also used in previously published research that correlated chest radiographic and chest computed tomography (CT) findings in pediatric COVID-19 pneumonia [5].

### Imaging technique

All chest radiographs were obtained as digital radiographs in anteroposterior (AP) (26, 13.9%) or posteroanterior (PA) (161, 86.1%) projection based on our department’s standard technique with chest radiography equipment. The AP projection radiograph technique included high voltage, tube current, and exposure times at a 100-cm focus-film distance with mobile radiography equipment (Digital Radiographic Mobile X-ray System; Shimadzu). The PA projection radiograph technique included high voltage, tube current, and exposure times at a 180-cm focus-film distance with mobile radiography equipment (Digital Diagnost; Philips Healthcare).

### Imaging study review

All chest radiographs were independently assessed by two radiologists (KMD and JK) with more than 20 years of expertise interpreting pediatric chest radiographs. The reviewers were blinded to all patient information, including clinical history, prior imaging studies, and original chest radiograph reports, included in this study population. The third reviewer (E.Y.L.), with 20 years of experience in interpreting pediatric chest radiography, served as a tie-breaker for discrepancies between the initial interpretations of the two reviewers without knowledge of the initial reviewers’ findings and patients’ clinical and imaging study information. A standard picture archiving and communication system (Cedara I-Read 5.2 P11; Cerner Image Devices) was used to review all chest radiographs included in this study.

### Imaging study evaluation and categorization

The reviewers first reviewed the chest radiographs for abnormalities according to the “Fleischer Society: glossary of terms for thoracic imaging” for abnormalities in the (1) lung parenchyma and airway (ground-glass opacity [GGO], consolidation, peribronchial thickening); (2) pleural (pleural effusion and pneumothorax), and (3) mediastinum (lymphadenopathy, pleural effusion, and pneumothorax) [22].

Subsequently, the chest radiographs were categorized into two groups: (1) group 1 (without radiographic evidence of COVID-19 pneumonia) and (2) group 2 (with radiographic evidence of COVID-19 pneumonia) based on the criteria for radiographic findings of COVID-19 pneumonia in children from an international expert consensus statement on chest imaging in pediatric COVID-19 patient management [6].

The grades of lung involvement were estimated using the chest radiographic score. Each lung was divided into three zones, and the participation of each zone was assessed. A score of 0 (normal) to 4 (full involvement of one zone) was ascribed to the development of COVID-19 lesions within each lung zone; a score of 24 showed total involvement of all six zones. The values for each of the six zones in each chest radiography scan were added together to provide a cumulative chest radiographic score ranging from 0 to 24, depending on lung parenchymal involvement [23].

### Evaluation of serum biochemical parameters

A focused review of the patients’ records was carried out to analyze the serum biochemical tests upon admission to the hospital. These investigations included (1) serum LDH; (2) C-reactive protein; (3) serum ferritin; (4) serum transaminases (aspartate aminotransferase [AST]); (5) alanine aminotransferase (ALT); (6) complete blood counts for platelet lymphocytes; and (7) white blood cell (WBC) levels.

### Evaluation of patient outcomes

The patients’ medical records were also reviewed for the length of hospital stay and patient outcomes. These were recorded independent of the treatment received by the patients.

### Statistical analysis

Descriptive statistics were obtained as means with standard deviations (SDs) for continuous variables. The minimum and maximum ranges were calculated for age. A frequency distribution with a percentage was calculated for categorical variables. Unpaired Student t-tests were used to compare the two groups (group 1 [without radiographic evidence for COVID-19 pneumonia] and group 2 [with radiographic evidence for COVID-19 pneumonia]) for normally distributed interval variables and the Mann–Whitney U test for non-normally distributed variables. Inter-observer reliability was calculated using Cohen’s kappa statistic (*k*) to assess the agreement between the two reviewers. Statistical significance was set at *p* < 0.05 (two-tailed). The patients’ medical records were carefully reviewed for the following serum biochemical parameters at presentation: (1) LDH; (2) C-reactive protein; (3) ferritin; (4) AST; (5) ALT; (6) platelet; (7) lymphocytes; and (8) WBC levels. Spearman’s rank correlation was used to evaluate correlations between the levels of the serum biochemical parameters and chest radiographic scores among patients. A simple binary logistic regression was used to investigate the importance of LDH in predicting abnormal chest radiographs. ROC analysis was used to examine the discriminating capacity of LDH in predicting chest radiographs, and the threshold was determined by maximizing Youden's index. The statistical software IBM SPSS 22.0 was used for the statistical analysis.

## Results

### Patient population

**Fig. 1:**
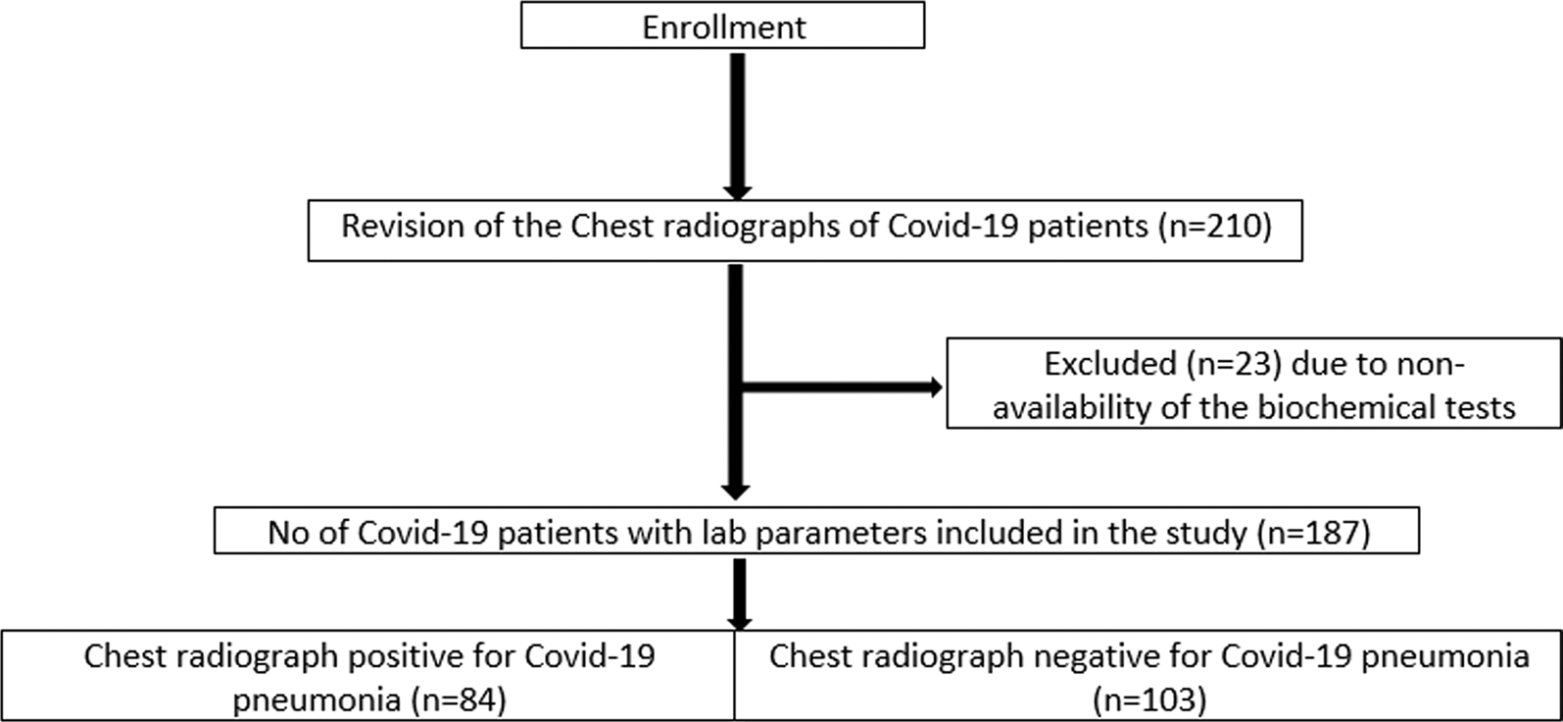
Schematic diagram of study information flow analysis. The initial chest radiographs of 187 patients were analyzed and divided into two parts (*N* = 84 and *N* = 103) depending upon the evidence of pneumonia

The final study population comprised 187 children (95 boys and 92 girls; mean age ± SD, 10.1 ± 6.0 years; range, nine months–18 years). Group 1 comprised 103 chest radiographs (55.0%) from 103 individual pediatric patients (54 boys and 49 girls; mean age ± SD, 12.5 ± 5.2 years; range, nine months–18 years). Group 2 comprised 84 chest radiographs (44.5%) from 84 individual pediatric patients (41 boys and 43 girls; mean age ± SD, 7.0 ± 5.6 years; range, 11 months–18 years). Of the 187 chest radiographs included in the final review and analysis, group 1 comprised 103 chest radiographs (55.5%), and group 2 comprised 84 chest radiographs (44.5%). A flow chart has been added for detailed explanation of the study (Fig. 1).

Among 187 patients, 103 patients (55.0%) presented with mild disease, 83 (44.4%) presented with moderate disease, and one remaining patient (0.6%) presented with severe disease based on the previously published study disease classification [2, 14]. There was no statistical difference in the duration of symptoms between the two groups (1.5 ± 1.2 in group 1 vs. 1.7 ± 1.0 days in group 2; *p* = *0.5*).

### Chest radiographic findings

**Fig. 2:**
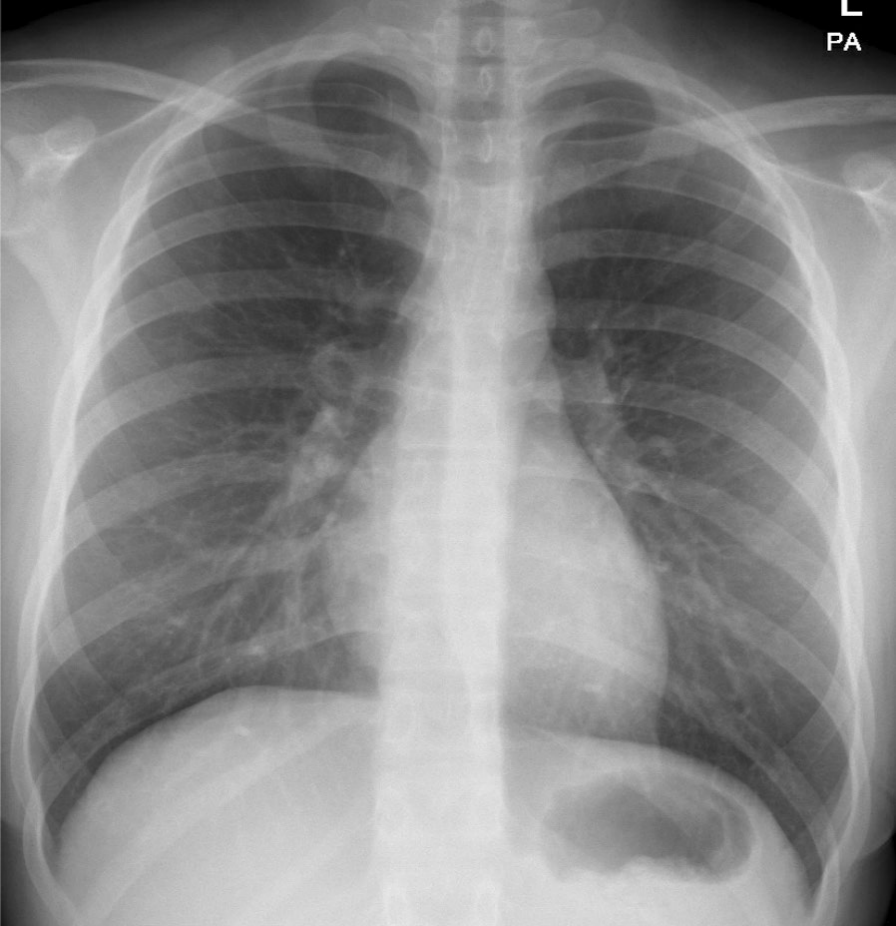
A 17-year-old girl with COVID-19 pneumonia based on positive RT-PCR test who presented with cough and rhinorrhea for 2 days. Frontal chest radiograph shows no radiographic abnormality. The patient’s serum biochemical parameters show a mildly elevated LDH level of 151 U/L

**Fig. 3:**
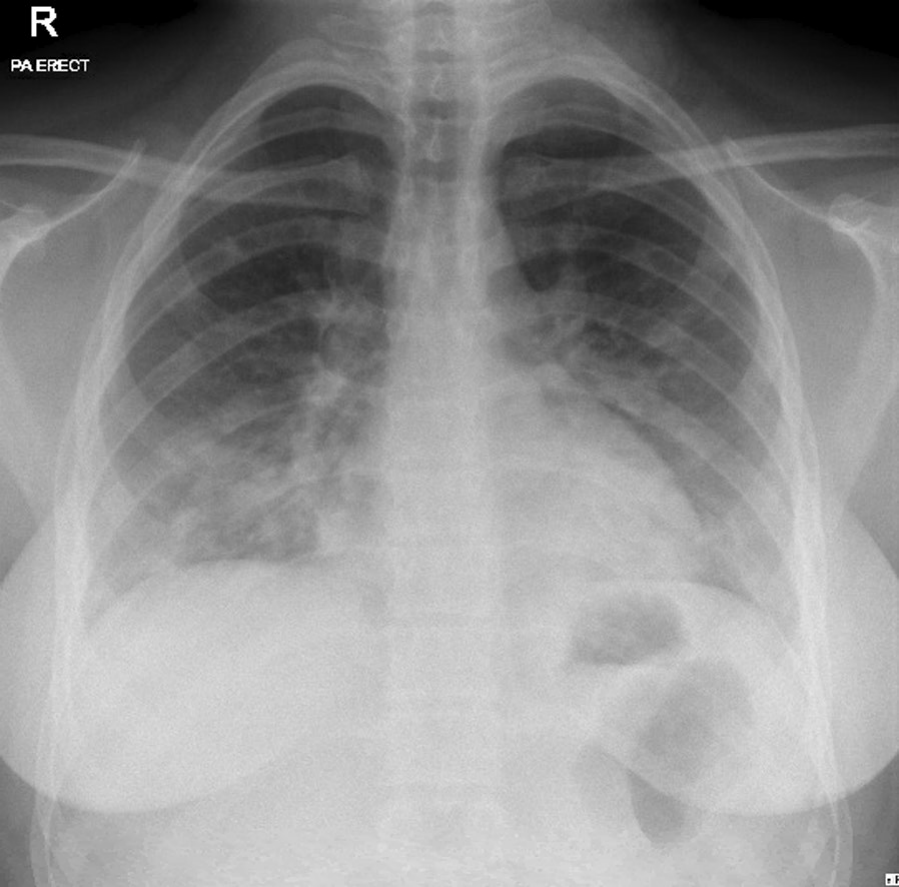
A 16-year-old girl with COVID-19 pneumonia based on positive RT-PCR test who presented with fever and cough for 3 days. Frontal chest radiograph shows bilateral multifocal ground-glass opacities and consolidations. The patient’s serum biochemical parameters show a significantly elevated LDH level of 260 U/L

The chest radiographic findings of patients included in this study are summarized in Table 1. No radiographic chest abnormalities were detected in group 1 patients (Fig. 2). The abnormalities observed on chest radiographs in group 2 included GGO in 75 patients (89.2%), GGO and consolidation in 6 (7.2%), peri-bronchial thickening in 1 patient (1.1%), consolidation in 1 patient (1.1%), and peri-bronchial thickening, GGO, and consolidation in 1 patient (1.1%) (Fig. 3). No pleural effusion, pneumothorax, or mediastinal lymphadenopathy was observed. The chest radiographic score varied from 0 to 6: 0 in 103 (55%), 1 in 33 (17.6%), 2 in 31 (16.6%), 3 in 12 (12.6%), 4 in 4 (2.1%), 5 in 3 (1.6%), and 6 in 1 instance (0.5 percent).

**Table 1:** Chest radiographic findings in 187 pediatric patients with COVID-19 pneumonia

Chest radiographic findings	Number (percentage) of studies
Normal CXR (Group 1)	103 (55)
Abnormal CXR (Group 2)	84 (45%)
*Types of abnormal CXR findings*	
GGO	75 (89%)
GGO and consolidation	6 (7%)
Peribronchial thickening	1 (1%)
Consolidation	1 (1%)
Peribronchial thickening, GGO and consolidation	1 (1%)

*CXR* Chest Radiography, *GGO* Ground-glass opacity

### Interobserver agreement on chest radiographic findings

Among the 187 chest radiographs reviewed between the two initial reviewers, there were discrepant interpretations in 3 cases, all related to GGO. The third reviewer, a tie-breaker, independently evaluated these three cases without knowing the initial reviewers’ interpretations or original report findings and determined that GGO was present in all three cases. There was an almost perfect interobserver agreement between the two reviewers for detecting the presence or absence of radiographic abnormalities in both groups (*k* = 0.96, *p* = *0.001).*

### Serum biochemical parameter findings and correlation with chest radiographic findings

The details of the patients’ serum biochemical parameters at presentation in group 1 versus group 2 are listed in Table 2. Group 2 pediatric patients (with radiographic evidence for COVID-19 pneumonia) had significantly elevated LDH (200.6 ± 53.4 vs. 258.9 ± 80.8 U/L, *P* = 0.001) with a moderate elevation of C-reactive protein (2.8 ± 5.0 vs. 5.7 ± 12.3 U/L, *P* = 0.05), serum ferritin (67.3 ± 93.8 vs. 93.2 ± 172.0 U/L, *P* = 0.25), and AST (24.8 ± 15.2 vs. 27.8 ± 12.0 U/L, *P* = 0.22) compared to the group 1 pediatric patients. There were no statistically significant differences in the remaining serum biochemical parameters.

**Table 2:** Comparison of Serum biochemical parameters in Group 1 (with Normal CXR) Versus Group 2 (with Abnormal CXR),

Serum biochemical parameters	Group 1 Mean ± SD	Group 2 Mean ± SD	*P* Value
LDH (60–100 U/L)	200.6 ± 53.4	258.9 ± 80.8	0.001
C-reactive protein (0.3–1.0 mg/dL)	2.8 ± 5.0	5.7 ± 12.3	0.05
Serum Ferritin (15–200 mg/L)	67.3 ± 93.8	93.2 ± 172.0	0.25
AST (0–35 U/L)	24.8 ± 15.2	27.8 ± 12.0	0.22
ALT (0–35 U/L)	20.2 ± 14.0	20.7 ± 17.2	0.86
Platelet count (150–350 × 109/L)	288.7 ± 80.2	304.2 ± 112.3	0.3
Lymphocytes (1.1–3.2 × 100 cells per L)	3.0 ± 2.4	3.5 ± 1.9	0.17
WBC (4–10 × 10 cells per L)	5.3 ± 2.0	5.4 ± 2.6	0.87

*CXR* Chest radiography, *SD* Standard deviation, *LDH* Lactate dehydrogenase, *AST* Asparate aminotransferase, *ALT* Alanine aminotransferase, *WBC* White blood cell

**Fig. 4:**
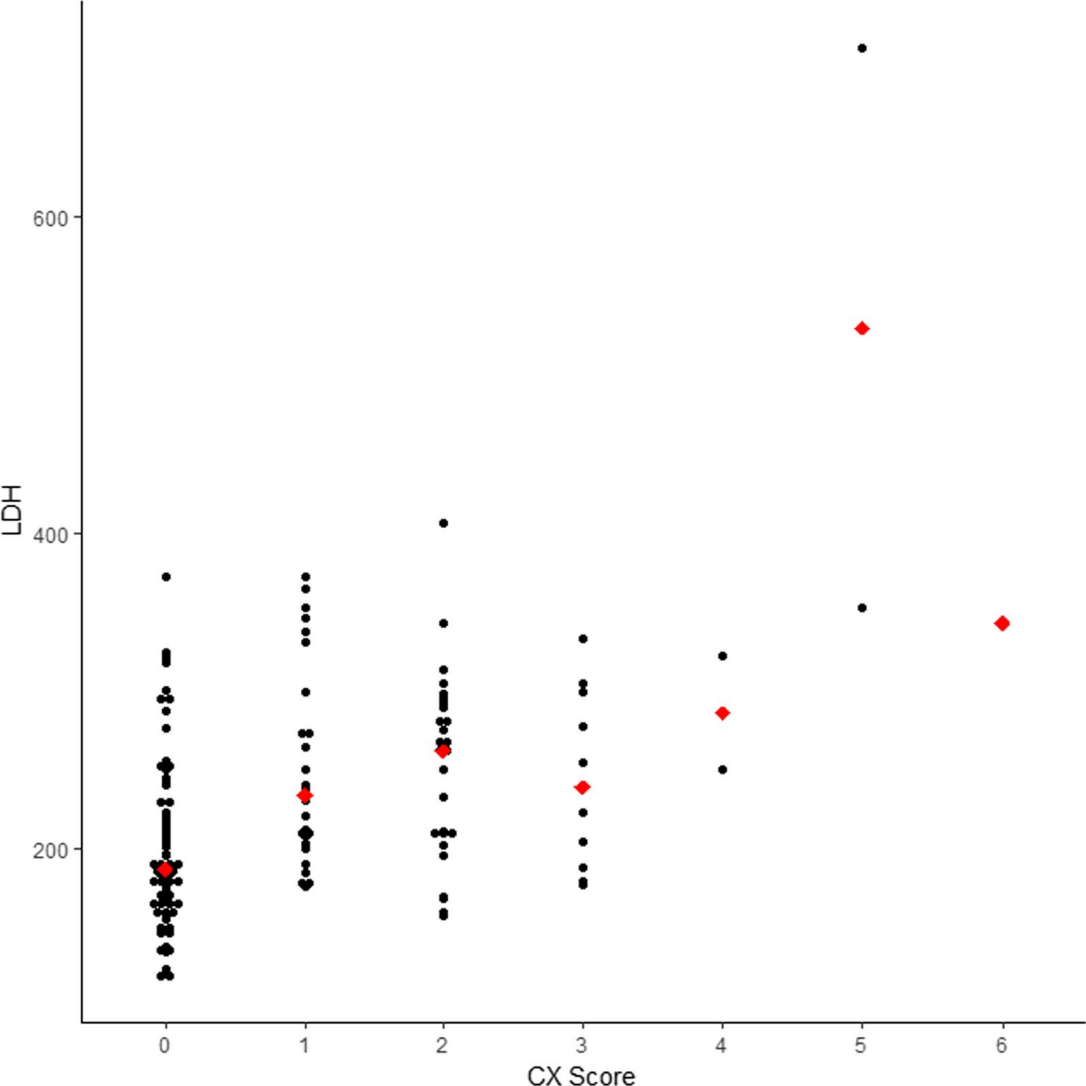
Dot plot of LDH by chest radiographic score. The red-colored diamond shaped observations in the dot plot represent the median of LDH in each chest radiographic score (CX score)

The Spearman’s rank correlation coefficient between LDH and chest radiographic score was computed, and the correlation value = 0.425 indicated that LDH and chest radiographic score had a significant relationship (Fig. 4). The remaining blood biochemical parameters associated with the chest radiographic scores were not statistically significant (Table 2).

**Fig. 5:**
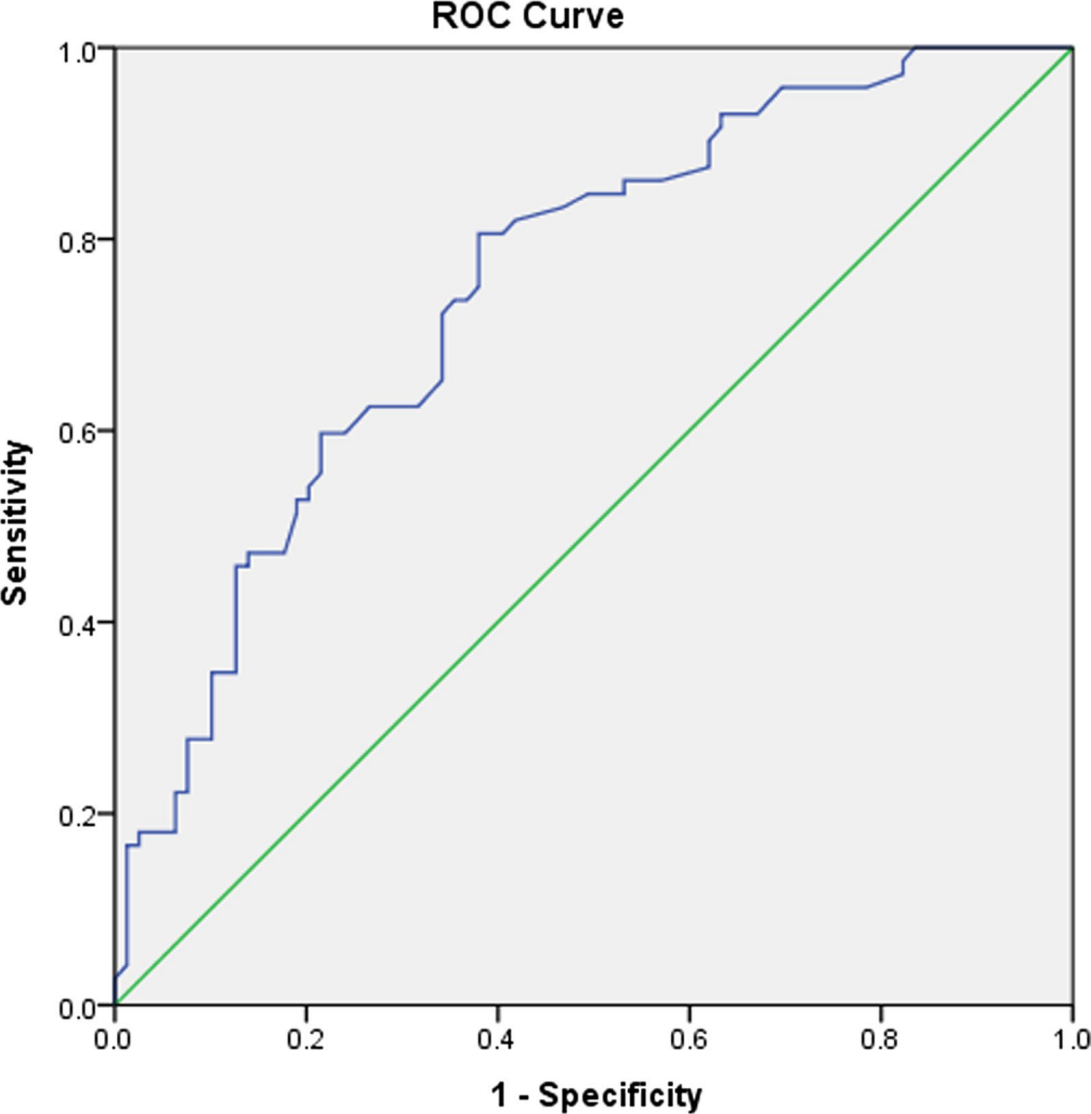
ROC curves for the discriminating ability of LDH (AUC = 0.75)

The significant differences found in LDH suggested it may predict abnormalities in a chest radiograph. With a *p*-value of < 0.001, the simple binary logistic regression showed that LDH is a significant predictor. With a sensitivity of 80.6% and a specificity of 62%, the ROC curve (Fig. 5) revealed that LDH > 200.50 U/L (Using Youden Index = 0.426) could predict abnormal chest radiograph with a sensitivity of 80.6% and a specificity of 62%.

### Patient outcome

The mean length of hospital stay was longer for group 2 patients (2.8 ± 3.4 days) than for group 1 patients (1.2 ± 1.0 days). The difference in hospital stay length between the two groups was significant (*p* = *0.001*). All pediatric patients in both groups completely recovered from COVID-19. After hospitalization, all pediatric patients were transferred to a quarantine facility for 14 days of isolation, including the days spent at the primary care hospital where the child initially arrived.

## Discussion

This study suggests that repetitive radiation-based imaging investigations can be minimized by assessing serum biochemical parameters such as LDH. Our study showed the effect of LDH on the prediction of abnormal chest radiographs (*p*-value of < 0.001) in a pediatric patient group of 187 patients and found that patients with elevated LDH > 200.50 U/L could predict abnormal chest radiographs with a sensitivity of 80.6% and a specificity of 62%. A simple binary logistic regression was used to explore the impact of LDH in predicting abnormal chest radiographs. ROC analysis was used to test the discriminating capacity of LDH in predicting chest radiographs, and the threshold was set by maximizing Youden's index.

In addition, the LDH result was strongly associated with the chest radiographic score in individuals with positive evidence of COVID-19 pneumonia. LDH levels were significantly increased (258.9 ± 80.8 U/L, *p* = *0.001*) in group 2 patients with pneumonia and almost doubled (200.6 ± 53.4 U/L) in group 1 patients without COVID-19 pneumonia on chest radiographs. As a result, we believe that serum LDH changes may be a better predictor of the body's pathological response to the COVID infection than chest radiographs, which were normal in 55% of our cohort despite LDH levels nearly twice the normal range.

It is well recognized that interpreting pneumonia on pediatric chest radiographs is subject to significant intra- and interobserver variability, with the majority (93.3%) of abnormalities found on chest CT being missed on chest radiographs [5]. Chest radiographs are associated with potentially harmful ionizing radiation exposure and often result in findings that are non-specific for the diagnosis of COVID-19 pneumonia in children [24]. Consolidations, increased central peribronchovascular markings, and GGO were the most frequently seen abnormalities on chest radiographs of COVID-19 pediatric patients, comparable to other lower airway inflammations and viral infections. Most of these observations may be non-specific, and chest radiographs cannot distinguish COVID-19 from any other pediatric lung infection [24]. The biochemical changes precede the imaging changes in the chest in the COVID-19 infection [25]. The radiological changes in chest imaging in patients infected with COVID-19 are based on the underlying diffuse alveolar damage (DAD) pathology, and vascular damage and thrombosis are prevalent in the COVID-19-infected lung[25]. In an autopsy series, the researchers confirmed that significant pulmonary vascular damage occurred in radiographically normal lung areas and histologically normal pulmonary parenchyma [25]. This is consistent with previous findings that vascular damage occurs prior to the formation of DAD in symptomatic COVID-19 infections [26]. Similar vascular damage (including endothelial injury and microthrombi) along with cytokine-mediated tissue injury has been documented in patients with acute respiratory distress syndrome (ARDS) and was responsible for the release of LDH in COVID-19 patients [27]. LDH has recently been validated as one of the most predictive markers for diagnosing and prognosis of COVID-19 pneumonia [28].

Our observations corroborate those of previous studies. For example, Jian et al. observed comparable elevated levels of LDH in pediatric patients, which they thought to be a possible sign of COVID-19 [17]. Similarly, Du et al. observed that the value and positive rate of LDH were significantly greater in children than in adults in a study of 67 adult and pediatric COVID-19 patients [29]. Erat et al. investigated the link between imaging findings (e.g., chest radiographic and chest CT findings) and serum biochemical markers in pediatric patients with COVID-19 pneumonia. They discovered a substantially higher LDH level (≥ 265 U/L) in patients with positive evidence of COVID-19 pneumonia on CT [20]. However, they did not compare LDH levels within the same group based on chest radiography findings. In another study of 237 COVID-19 pediatric patients, the LDH level was increased ≥ 280 U/L in 43% of cases with definite evidence of pneumonia in an imaging study [30]. However, LDH comparisons between chest radiography and CT have not been performed. The smaller sample size and ethnic variance are most likely to blame for the relative heterogeneity of LDH levels in other series, including ours. For the most verified level of LDH, a higher sample size would be necessary.

During the initial phase of COVID-19 infection in China, chest radiography and chest CT are widely used as initial screening tools to evaluate disease severity in most COVID-19 patients, including children [31]. Most pediatric experts believe that chest radiography can diagnose airway infection or pneumonia in symptomatic children and that CT should be used only when there is a clinical reason to be concerned, particularly in children with coexisting medical problems [24]. The radiation dosage for a chest radiograph in a single exposure may be minor; pediatric patients often need repeated tests to monitor their health, resulting in relatively large cumulative doses [11]. Since infants and children have smaller body diameters and their organs are less protected by surrounding tissues, the dosages to their internal organs are more significant than those for adults [12]. Additionally, metabolism and physiology change with age, affecting the radionuclide concentrations in various organs and the dosage delivered to those organs for a given intake [11]. Cancer risk increases linearly with lower dosages without a threshold, and even the lowest dose can produce a minor increase in risk in humans [13]. In situations like medical exposure, infants and children may get much larger radiation doses than adults if the technical parameters are not correctly calibrated [11]. Hence, children are far more susceptible to the carcinogenic effects of ionizing radiation than adults, and children have a longer life expectancy, creating a greater window of opportunity for radiation harm to manifest [13]. According to the American College of Radiology Appropriateness Criteria, imaging is not required in well-appearing immunocompetent children ≥ 3 months of age who do not require hospitalization [32].

Point-of-care ultrasound (POCUS), which is rapid, portable, reproducible, and non-ionizing, has increased in pediatrics population in recent years. Using specific lung ultrasound (LU) patterns in the differential diagnosis and prognosis of pneumonia and acute bronchiolitis have been discovered to be beneficial. The first case series reporting the key ultrasonography findings in children with confirmed COVID-19 pneumonia was recently published, implying pulmonary involvement similar to previous viral infections [33]. Patients diagnosed with COVID-19 and with a higher LDH threshold may also benefit from this technique. A similar strategy could significantly impact the safer diagnosis and follow-up of COVID-19 patients without exposing them to chest radiography radiation.

Our study had two major limitations. First, the study population was relatively small. However, we want to emphasize that our retrospective study comprised a unique group of pediatric patients who had chest radiographs and serum biochemical parameter results obtained on the same day during initial admission to the hospital. Second, our study population had only a single frontal chest radiographic view without a lateral chest radiographic view, which may have limited evaluation, especially the ability to detect small pleural effusion or mediastinal lymphadenopathy. Previous studies show that pleural effusion and mediastinal lymphadenopathy are rarely seen in pediatric patients with acute COVID-19 pneumonia [3–6]. Therefore, we believe that including only frontal chest radiographs most likely did not substantially affect our study results. Additionally, we would like to underline that single frontal chest radiographs are frequently conducted to minimize ionizing radiation exposure.

In conclusion, our data show that, when compared to other biochemical markers, LDH might predict abnormal chest radiographs in a pediatric patient with confirmed COVID-19 infection with an 80.6% sensitivity and a 62% specificity. Additionally, it underlines that the pathological response to COVID-19 infection in the chest may be better detected early on by biochemical parameters rather than chest radiological imaging. As a result, we postulated that LDH levels might be potentially used as a substitute for chest radiography in children with COVID-19, hence minimizing radiation exposure. Additionally, segmenting patients according to their LDH levels may help expect having a long hospital stay, which relaxes the hospital system, saves costs for patients and hospitals, and dramatically reduces caregiver stress, enhancing caregiver well-being.

## Data Availability

The datasets used and/or analyzed during the current study available from the corresponding author on reasonable request.

## Abbreviations

LDH: *lactate dehydrogenase*
*AST*: Aspartate aminotransferase
ALT: Alanine aminotransferase
RT-PCR: Reverse transcriptase-polymerase chain reaction
GGO: Ground-glass opacity
hs-CRP: High sensitivity C-reactive protein
CXR: Chest radiography
SD: Standard deviation
WBC: White blood cell
